# Predictive Processing and the Varieties of Psychological Trauma

**DOI:** 10.3389/fpsyg.2017.01840

**Published:** 2017-10-17

**Authors:** Sam Wilkinson, Guy Dodgson, Kevin Meares

**Affiliations:** ^1^School of Philosophy, Psychology and Language Sciences, University of Edinburgh, Edinburgh, United Kingdom; ^2^Northumberland, Tyne and Wear NHS Foundation Trust, Newcastle upon Tyne, United Kingdom

**Keywords:** predictive processing, Bayesian brain hypothesis, trauma, nervous system, PTSD, psychosis

## Abstract

A recently popular framework in the cognitive sciences takes the human nervous system to be a hierarchically arranged Bayesian prediction machine. In this paper, we examine psychological trauma through the lens of this framework. We suggest that this can help us to understand the nature of trauma, and the different effects that different kinds of trauma can have. We end by exploring synergies between our approach and current theories of PTSD, and gesture toward future directions.

## Introduction

Psychological trauma, often encountered under the diagnostic category of post-traumatic stress disorder (PTSD), occurs in a wide variety of contexts and has a variety of symptomatic presentations (see, e.g., Bisson et al., 2015). In this paper, we suggest that, if we view the nervous system as a hierarchically arranged Bayesian prediction machine, we can better understand the nature of psychological trauma, and the different effects that different kinds of trauma can have on the human mind. In particular, we will demonstrate how such an approach can help us to shed light on three things. The first is the nature of the phenomenon known as “dissociation.” The second is the underlying nature of the difference between what has been called “Type 1” and “Type 2” trauma (now a widely used distinction first introduced by Terr, 1991). The third concerns the fact that trauma is not only associated with PTSD, but also with psychosis. We suggest why this might be so, and why certain types of trauma, at certain periods in the life-course, might be more likely to lead to psychosis than to PTSD. We end by exploring synergies between our approach and current theories of PTSD, and point toward future clinical and research directions.

## What Do We Mean by PTSD?

Post-traumatic stress disorder, like so many diagnostic categories, is broad and heterogeneous. In the most general sense, PTSD occurs when a traumatic one–off event or extended period leaves its mark on the human nervous system, giving rise to undesirable symptoms. These symptoms vary (see, e.g., American Psychiatric Association, 2013), but include disturbing and intrusive thoughts, feelings, or dreams related to the traumatic phenomena, distress in the presence of trauma-related cues, attempts to avoid trauma-related cues, alterations in how a person thinks and feels, heightened arousal, and hyper-vigilance^1^. A particularly well-known symptom of PTSD, probably in part because it captures the public imagination, involves vivid recollections or re-livings of the traumatic events, which are often referred to as “flashbacks.”

We have spoken not only of traumatic *events* but also of *extended periods* of trauma. This corresponds to two broadly different kinds of stressor that tend to be associated with PTSD (Terr, 1991; Courtois and Ford, 2009). The first, which is perhaps the most commonly appreciated, involves a one–off and usually life-threatening event of cataclysmic significance. This is sometimes referred to as “Type 1 trauma.” The second involves an extended period of exposure to trauma, often in the form of heightened perceived threat. Here the lasting psychological impact comes from a temporally extended need to stay vigilant in the face of an ongoing expectation of impending harm. This occurs, for example, during extended periods of childhood or domestic abuse, or during extended periods as a combatant in a war zone (Doctor and Shiromoto, 2009). This is sometimes referred to as “Type 2 trauma”^2^.

## The Predictive Processing Framework

Most accounts of PTSD (some of which we will examine later) start with the symptoms of PTSD and try to explain them. Our methodology is importantly different. We start with a framework for thinking about the human nervous system, and think about how, in the light of this framework, trauma, and different types of trauma, might affect the human mind. We proceed on the basis that a good current understanding of the nervous system is captured by the predictive processing framework (PPF) (Friston, 2005; Clark, 2013; Hohwy, 2013). Adopting such a framework paints an accurate picture of the effects that trauma has on the nervous system, and also points us toward exciting future theoretical and clinical directions.

The application of the PPF to various mental disorders and conditions is not new. For example, it has been applied to psychosis (Fletcher and Frith, 2009), auditory verbal hallucination (Wilkinson, 2014), autism (Pellicano and Burr, 2012), hysteria (Edwards et al., 2012), and stress (Peters et al., 2017). To our knowledge, it has not been applied to psychological trauma.

At this point it is important to clarify that we view the PPF as precisely that, namely, a framework [see Wilkinson (2014) for an explanation of the difference between frameworks, theories, and models]. By this we mean that it is a useful way of thinking about a certain domain of interest, which is one of many other potentially equally valid and useful ways of thinking. The nervous system doesn’t literally make predictions, draw inferences, or select hypotheses [strictly speaking, only persons as a whole literally do these things (Ryle, 1949)] but it can be helpfully *thought of* as doing these things. Our commitment to this sort of pragmatism is reflected in our final section, where we see our proposals as complementing, rather than conflicting with, existing accounts of PTSD.

### The Information-Processing Framework

A great deal of work within the cognitive sciences has taken place within a very broad framework that we might call the “information-processing framework” (Broadbent, 1958). This does not pick out any particular theories or models, but forms a broad background assumption about the fundamental nature of cognition, against which such theories or models are built [Marr’s (1982) account of vision is one canonical example]. According to this framework, the nervous system’s main task is to process information from the outside world. This information comes in the form of impacts upon our sensory receptors. When such information arrives, it gets passed along a processing chain, which, stage by stage, processes the information until there is something that corresponds to our rich perceptual experience in all its complexity and sophistication.

There is a huge amount of variation among the theories and models that fall within this information-processing framework. For example, many models don’t think of processing as only being “bottom-up,” but also allow for various “top-down” influences [see Neisser (1976) for an early example of this]. It is therefore inaccurate to say that the framework is characterized by a uniquely bottom-up direction of processing. What does, however, characterize the information-processing framework concerns what the relevant causal and temporal antecedents of a given perceptual experience are. The information-processing framework will say that the relevant antecedents are environmental events, and information from those events. Models or theories that allow top-down influences simply say that these impacts on how that incoming information is received, processed, and passed on. Top-down influences notwithstanding, any given perceptual experience is preceded by the relevant environmental event (which the experience is often thought to somehow represent) and constructed out of information from that event.

Two caveats are in order at this point. First, the focus in presenting the information-processing framework has been on perception and the processing of information from the environment. There are, within the information-processing framework, accounts of all aspects of cognition, and not just perception (e.g., action, memory, imagery, etc.). However, perception is the simplest for the purposes of presenting the framework. The second caveat is that we are not primarily seeking to criticize the information-processing framework, but rather wish to use it as an illustrative contrast against which to present the PPF that we will be working within.

Useful though it may be, the information-processing framework has some shortcomings. First of all, it requires the nervous system to wait for events in the world to happen, and for information from those events to reach it. One might expect the nervous system to have evolved to be more pro-active, more anticipatory. Secondly, according to the framework, the information that comes in early on in processing is relatively noisy, and then, as it gets passed on, it gets more and more complex and fine-grained. Given that on this model the information at each stage is explicitly represented in neural activity, this means that, as we go up the processing chain, things get progressively more and more complicated, and more and more costly in terms of the using of neural resources. One might expect the nervous system to be more efficient in its use of resources. There is an alternative framework that presents the nervous system as both pro-active and cost-effective. This is the framework we present now.

### The Predictive Processing Framework As an Alternative

There is a different way of thinking about what the nervous system does, and about the fundamental nature of its relationship to the environment. Although this framework has recently picked up many adherents, its roots go back at least as far as von Helmholtz (1866/1962).

Instead of thinking of the nervous system as responding to – and constructing experience out of – impressions on the nervous system, we can instead think of it as actively predicting (or attempting to predict) rather than passively responding to sensory information. Conscious experience, on this view, is not constructed out of sensory information after the relevant event, but is constituted by your nervous system’s best predictions, and thereby stays one step ahead of what’s happening in the outside world. This of course is not to say that the environment plays no role: it plays a crucial, but somewhat different one. It keeps our predictions in check; it keeps them accurate and world-directed. Thus, experience, though massively flexible (and more flexible than in the information-processing framework) isn’t an “anything goes” situation; it is still, at least usually (hallucinations and delusions aside), answerable to what is going on in the world.

To understand the PPF in more detail, it is worth reflecting on two things: *ambiguity* and *efficiency*; in particular, the ambiguity present in (even the cleanest) sensory signals, and the fact that the nervous system has to operate as efficiently as possible.

The nervous system’s main task is to settle on a hypothesis about what is going on, based on *ambiguous input*. This ambiguity means that a number of different hypotheses are compatible with the input (and, indeed, the same hypothesis is compatible with different inputs). Given this, how does the nervous system settle on one hypothesis rather than another? It needs to take something else into account: not just the fit of the hypothesis with the input, but also how statistically likely that hypothesis is, independently of that particular input. This is called the “prior probability” of the hypothesis, and will often, but not always (it is arguably sometimes a function of innate biases), be assigned as a result of past experience. As a result, a hypothesis could fit the input extremely well, but its prior probability could be so low that it isn’t even considered. Conversely, a hypothesis could have such a high prior probability, that, even though it doesn’t fit the input well, it is settled upon. What we have just described is Bayesian inference, and the PPF can be helpfully thought of as a neural implementation of this Bayesian strategy.

One nice illustration of a case where a hypothesis has such a high probability, but doesn’t fit the input very well, is the case of the Hollow Mask Illusion. When you are presented with a rotating mask that is slowly turned to present you with the concave back of the mask, your nervous system “corrects” the concave stimulus into a convex stimulus. You experience the concave back of the mask as convex. This is due to the “convex face hypothesis” having such a high prior probability that it over-rides the incoming signal. This is because faces are extremely important stimuli, and your nervous system has the expectation that the faces you will encounter will always be convex. The prior probability of the “convex face” hypothesis is so high that, even though the “concave face” hypothesis would better match the input, it is never selected.

As a result of selection pressures, the nervous system will implement this disambiguation strategy as *efficiently* as possible. “Efficiently,” in this instance, means performing the inference as quickly, as accurately, and with as little energy expenditure as possible. Of course, speed, accuracy, and energy expenditure tend to pull in opposing directions. The faster you go, the less accurate you are likely to be and the more energy you will expend. Again, if you are trying to be too accurate, you will take too long, and expend more energy than is necessary, and so on. As a result of this, it is a matter of finding an optimal trade-off between these three desirables [see Peters et al. (2017) for an account of when this trade-off fails in cases of stress].

For now, it is important to note that the way in which the Bayesian strategy is implemented in the nervous system is highly efficient, and this is where the notion of prediction comes in. Predictive strategies are used to maximize efficiency in informatics. In fact, it forms the basic principle of data compression. You save on bandwidth, when passing a message, by only passing on what is newsworthy. What counts as “newsworthy” is simply what the receiver of the message hasn’t already predicted, namely, prediction error. Of course, what counts as the “sender” and “receiver” of the message in the case of the nervous system are predominantly *within it*, namely, they are different parts of the nervous system, of which the most substantial part is of course the brain.

This counts as an implementation of a Bayesian strategy insofar as the selection of a hypothesis determines a set of predictions about subsequent inputs, namely, inputs that are compatible with the hypothesis. If the hypothesis predicts inputs well, it will be kept. If it predicts them badly, it will be tweaked or abandoned altogether in favor of another hypothesis. In other words, one hypothesis is selected rather than another if it better *minimizes prediction error*.

### Two Further Developments: Precision and Hierarchy

There are two important ways in which the PPF can be developed further. One concerns “precision weighting,” and the other the “hierarchical arrangement of hypotheses.” We take these in turn.

#### Precision Weighting

Although incoming signals are ambiguous, in different contexts, the degree of ambiguity will differ. To maximize its predictions, the nervous system needs to accurately estimate how much ambiguity (uncertainty) there will be. In other words, it needs to make second-order predictions, namely, predictions about how much it should rely on its predictions (which amounts to how much it should pay heed to the prediction error).

In contexts where low ambiguity is expected (high signal-to-noise ratio), higher precision will be estimated, and the prediction error will be taken more seriously. Conversely, when there is high ambiguity (or, through some top-down influence, there is low interest in the stimulus), low precision is estimated, and the prediction error will be taken less seriously. This is called “precision weighting,” which amounts to turning up (or down) “the gain” on prediction error, and is taken to be modulated by neurotransmitters such as dopamine (Corlett et al., 2010). The role of precision weighting has taken center-stage in many PPF accounts of psychopathology (see, e.g., Edwards et al., 2012; Adams et al., 2013). Within this framework, turning up precision corresponds to increased attention, i.e., attending selectively to precise, newsworthy information. As a result, these accounts take pathological symptoms to be the result of attentional abnormalities under this somewhat broad and neurobiologically informed construal of attention (Hohwy, 2012).

#### The Hierarchical Arrangement of Hypotheses

The second important further development of the PPF is that these hypotheses are *hierarchically organized*. They are hierarchically organized in two different but complimentary senses. The first sense is that the hypotheses of one level provide the inputs for the next level (for evidence of hierarchical processing in the brain, see Felleman and Van Essen, 1991). The second is that the nature of the hypotheses differs depending on where they are in the hierarchy. “Higher” parts of the hierarchy are, roughly, those parts that are further away from the sensory stimulus. These tend to be at a lower temporal timescale, and a higher level of abstraction. They might correspond to abstract generalizations, such as the belief that lions are dangerous. “Lower” parts of the hierarchy are closer to the sensory stimulus. These tend to be at higher temporal frequencies, and at low levels of abstraction. These, for example, correspond to early stages of visual processing: your nervous system’s early statistically driven attempts to make sense of noisy inputs. Of course, in order to express these neurally encoded predictions we need to use rough-and-ready descriptions in natural language (in this case English; “Light tends to come from above”/“This is a face”), but there is nothing linguistic about the priors/hypotheses themselves.

**FIGURE 1:**
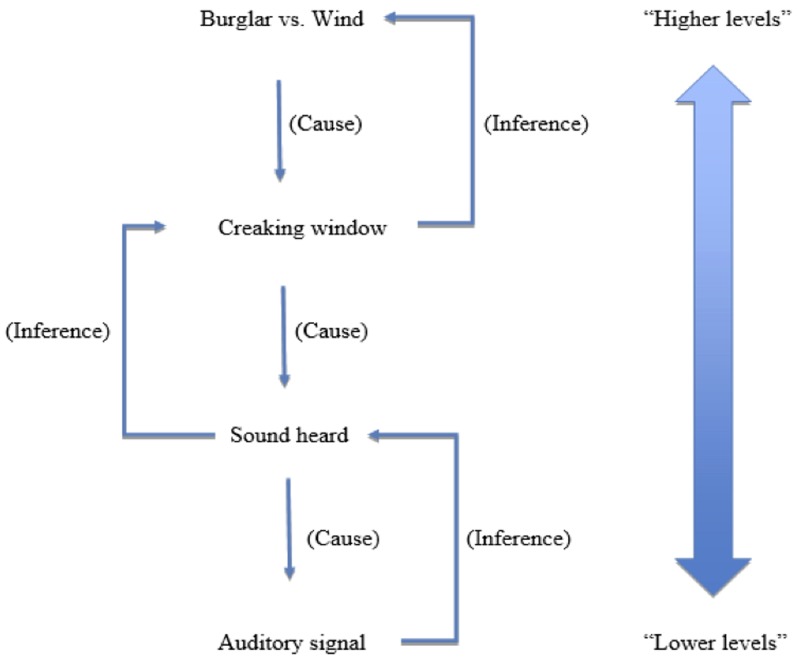
A schematic representation of a predictive processing hierarchy.

Let us take an example [adapted from Pezzulo (2013)] to illustrate the predictive hierarchy. Suppose that, on the basis of a noise, which you take to be a squeaking window, two hypotheses present themselves about what is going on: either the wind blew the window, or a thief is clambering into your house. At the stage where those two hypotheses are competing, a great deal of ambiguity has already been resolved, in a Bayesian fashion, at lower levels of the hierarchy. This can be represented in the **Figure 1**.

Schematically put, the low-level sensory information is noisy and ambiguous, so lower levels of the hierarchy select hypotheses that determine what the sound is like qualitatively speaking (its loudness, pitch, and timbre)^3^. These features themselves remain ambiguous, and so, slightly further up the hierarchy, there is a further disambiguation about what the sound is (its significance, what caused it), and so the hypothesis that it is a squeaking window rather than, say, a chirping bird, is selected. Once the “squeaking window” hypothesis has been settled upon, there may be further disambiguation regarding the significance of the squeaking window: was it a burglar, or was it the wind? This hypothesis, like the hypotheses lower in the hierarchy, will be selected in a Bayesian manner. Both hypotheses have a good fit (it they were correct, they would produce the “squeaking window” sound), but they may have different prior probabilities. For example, if the crime rates in the area are very low, and you know it is a windy night, the “wind” hypothesis would have a greater prior probability, and would be selected.

### Some Important Consequences

The PPF has some important consequences for our understanding of trauma and its effects on the human mind. Firstly, according to the PPF, conscious experience is much less reliant on input. Indeed conscious experience is simply a function of your nervous system’s best hypothesis, so, in principle, doesn’t *need* any input at all (indeed we see this not only in hallucinations, but also in dreams and in imagery and imagination). The role of input, as we have seen, is only to keep the nervous system’s hypotheses on track. This means that when we come to explain experiences that correspond badly to what is going on in the world, like hallucinations, we don’t so much need to ask ourselves, where did all of this input (or the illusion of this input) come from? Instead we ask: why did the nervous system adopt this erroneous hypothesis? And because our nervous system’s hypothesis building is largely self-generated, and only kept accurate by a very delicate process of correction, this doesn’t only make the explanatory task more palatable, it also has a welcome normalizing effect. The nervous system of a normally perceiving organism and the nervous system of a hallucinating organism are much more similar to one another within the PPF than within the information-processing framework. Indeed, according to the PPF, perception *just is* controlled hallucination. All of this, which applies to a better understanding of hallucination (Wilkinson, 2014), also applies to flashbacks. Indeed, as we are about to see, flashbacks can be understood as your nervous system generating a very specific hypothesis in contexts where it is inappropriate to do so.

A second vital consequence of the PPF does not pertain to symptoms of trauma, but rather to how trauma affects the nervous system. According to the PPF, processing and learning (e.g., perception and perceptual learning) are two sides of the same coin. There is no sharp division between a programming phase and a deployment phase. Our nervous systems “learn on the job,” so to speak.

On many standard models, especially classical computationalist ones, the nervous system is like a computer: the processing and the learning are kept very separate. Therefore, trauma can only be understood as an event that has been so extreme to process, that it has somehow broken the system. One upshot of this is that traumatic experience and non-traumatic experience are radically different in kind: while the latter uses the system, the former breaks it. The PPF, on the other hand, views all encounters with the world as involving learning to some degree. It is just that a traumatic experience involves a great deal of learning for a very specific context that is maladaptive in the vast majority of contexts that the person is likely to find herself in. But the fundamental process of a traumatic and non-traumatic experience is fundamentally the same^4^.

Another upshot of the PPF is that it can account for why, in different phases of the life-course, the nervous system is more susceptible to trauma. As we just said, processing and learning are two sides of the same coin: our nervous systems learn on the job. But, they tend to learn more, and more rapidly, the earlier on in the job they are, since they are laying down priors anew. Therefore, the younger someone is when they are subjected to trauma, the more profound and generalized the (in this case maladaptive) learning will be. This idea will become very relevant when we come to examine post-traumatic psychosis.

Finally, and in a related manner, the PPF gives us the tools, with its central notion of hierarchy, to make sense of when the hypothesis-selection of someone’s nervous system will simply give rise to a low-level sensory anomaly, or when, at the other extreme, it will constitute the subject’s world-view.

## Predictive Processing and Trauma

Not only does the PPF have the means to explain the effects of traumatic experience on the nervous system more generally, it also has the structural resources to explain the differing effects of different kinds of trauma. The differences we present (which we do not take to be exhaustive by any means) are, first, between Type 1 and Type 2 trauma, and, second, between PTSD and trauma-based psychosis. But first, we show how it can help to account for a phenomenon that is central to trauma, namely, dissociation.

### Dissociation

It is widely accepted that trauma can lead to dissociation [see, e.g., van der Kolk and Fisler (1995), Putnam et al. (1996), and many more], and that dissociation is in turn associated with a number of mental disorders, including psychosis, PTSD, depersonalization disorder (DPD) (see, e.g., Moscowitz et al., 2008). The concept of dissociation is intended to capture a certain detachment or disconnection that someone might feel toward their current perceptions and emotions, or toward their experiences of the world around them more generally (Lynn and Rhue, 1994). In relation to trauma, dissociation comes in two very different varieties: *peritraumatic* and *post-traumatic* (Ursano et al., 1999). Peritraumatic dissociation consists in a detachment from what is happening during a traumatic event [it can also lead to amnesia with regards to that event (e.g., Freyd, 1994)]. Post-traumatic dissociation consists in a general and more long-lasting sense of detachment from the world in the aftermath of a traumatic event. In extreme cases, this leads to a diagnosis of DPD.

Dissociation in general, and these types of dissociation in particular, can be nicely accommodated within the PPF. The crucial notion within the PPF for this purpose is the notion of hierarchy. To state the account plainly, and subsequently flesh it out: dissociation involves a disconnection of the lower levels of the hierarchy from the higher levels.

Recall that the lower levels of the hierarchy deal with more sensory and concrete happenings in the world, and operate at faster timescales. Thus, at these lower levels your nervous system selects hypotheses about what is happening in terms of shapes, colors, sounds, and at mid-levels, in terms of objects and events. However, an extremely important feature of the overall multi-level hypothesis that constitutes a normal experience is not just that this is all happening, but it is happening to *you*. Thus, built into the overall hypothesis is a self-model. How this self-model is constructed and fits into your nervous system’s dynamic hypothesis construction is currently being explored [see Letheby and Gerrans (2017) and Millière (2017) who both examine ego dissolution from within the PPF]. Whatever the finer details, it would certainly be very high up in the hierarchy: a-modal, highly abstract, temporally extended over long timescales, etc. If the prediction-error (bottom-up signaling) from the low-level and mid-level hypotheses is blocked or down-modulated, the resulting experience will be one where something is happening (colors and sounds at the lower levels, objects and events at higher levels, etc.), but it is not experienced as happening to *me*. If this is short-lived and occurs only during a traumatic event, then what we are talking about is peritraumatic dissociation. If this is a general state of affairs that is brought to bear on the experience of the world generally, then we are talking about post-traumatic dissociation, and potentially a dissociative disorder (or even depersonalization).

This is what dissociation looks like within the PPF, but why would it occur? Most models of peritraumatic dissociation build on the classic work of Janet (1904) who claimed that dissociation was a protective mechanism, allowing traumatic experiences and memories to be compartmentalized and thereby minimize distress (see, e.g., van der Hart et al., 2010). We think that this is undoubtedly along the right lines. Within the PPF, distress, namely, the subjective experience of strong negative affect, is to be understood as the generation of a hypothesis about what is happening to oneself that is mainly designed to minimize prediction error from interoceptive sources (namely, bodily change) (see Seth, 2013). One way to minimize distress would be to hypothesize that the events are not happening to oneself. Then, in line with this, the relevant bodily changes are also not hypothesized to be taking place, and, as a result, the negative affect is then heavily, or perhaps even entirely, mitigated.

So far this is in keeping with the vast majority of accounts of why dissociation takes place (see Dell and O’Neil, 2009). But such accounts view the natural drive toward minimizing negative affect as so self-evident (indeed, even trivial) that this amounts to an explanatory bedrock. Within the PPF we can take things one step further by reflecting on the biological nature and purpose of negative affect. It is to motivate the organism to get rid of it, to make it act so as to improve its current situation (Van de Cruys, 2017). Thus, the PPF’s re-framing of the dissociative mechanism is not just about the negativity of the event, but also about one’s helplessness in the face of the event in question [see Peters et al. (2017) for an account of stress within the PPF along these lines]. The negative affect is quarantined (and ultimately reduced/eliminated) through dissociation when it is clear that it can no longer be of help to you: it cannot motivate you to get out of the current situation because it is clear that you can’t get out of it^5^.

One outstanding question is: What makes dissociation persist in cases of post-traumatic dissociation? The more obvious answer is to say that we are talking about a similar state to the state of peritraumatic dissociation, but where the drive toward distress-minimization (self-protection) is extended through time. Everything the dissociated subject experiences (and not just the traumatic event) is happening but is not happening to them. A less obvious answer would be to say that post-traumatic dissociation is, in spite of superficial similarities, different in its underlying nature from peritraumatic dissociation. Perhaps, for example, the self-model is not disconnected from lower levels of the hierarchy, but has somehow been broken or disintegrated. The possibility of this happening, and how it might happen, is at this stage highly speculative, but this is an interesting area for future research and theorizing [the work of Letheby and Gerrans (2017) and Millière (2017) on ego dissolution in the context of psychedelic drug-taking might offer some clues here].

### Type 1 vs. Type 2 Trauma

In Type 1 trauma, a single traumatic event of cataclysmic and usually life-threatening significance leaves its mark of person’s nervous system. The symptoms of Type 1 trauma tend to be distress in the presence, and avoidance, of cues that are a reminiscent of the event. Sometimes these environmental or even self-produced cues (e.g., patterns of thinking/imagery) yield vivid flashbacks (Brewin et al., 2012).

Here is how the PPF makes sense of Type 1 trauma. What happens, when the traumatic event is experienced, is that a multi-level hypothesis is selected which corresponds to the conscious experience of the traumatic event. Part of that hypothesis, that conscious experience, is its life-threatening significance. As a result, selecting that hypothesis is something that your nervous system just cannot miss in the future^6^. What this amounts to in Bayesian terms is a hypothesis that, due to its importance for survival, is given an unusually high prior probability. That biases the selection of that hypothesis such that it will be selected even when the fit with the input is relatively poor. And, as we saw, hypothesis selection determines the conscious experience at that time. That is why something merely reminiscent, even only vaguely so, can act as a trigger, and lead to a vivid reliving of the event. Thanks to the hierarchy, this needn’t be experienced as an actual reliving of the event. The higher-level hypothesis concerning where and when the subject is remains intact (and hence accurate) and therefore there is often an awareness that, although intensely unpleasant, and potentially highly disruptive, this is “just a flashback” (Brewin, 2015).

Matters are further aggravated since the perceived fit of the hypothesis is made stronger by interoceptive feedback loops. What we mean is that, not only is the hypothesis of supreme significance, it is also heavily affectively charged. Thus, it needn’t be that something reminiscent of the traumatic event be *directly mistaken* by your nervous system for the traumatic event itself. Rather, what will often happen is that the trigger will give rise to an interoceptive affective state that will itself need explaining along with the sensory evidence (Pezzulo, 2013). To take an example, if you return to **Figure 1**, suppose you have just seen a horror movie and are in a generalized state of fear. The “wind” hypothesis may have a higher prior probability, but the burglar hypothesis would now have a better fit, since it would not only explain the squeaking window, but also the fear that you feel. Similarly with trauma, the traumatic hypothesis has better fit since it not only explains (albeit poorly) the sensory evidence; it does a pretty good job of explaining your affective state too. This is a “feedback loop” because the trigger need only initially result in a relatively mild emotional state, which then promotes something closer to the trauma hypothesis, which in turn will lead to a stronger emotional state, which in turn will lead to a more determinate and negative hypothesis, etc. In short, there is a vicious cycle leading to progressively more powerful affective states, and progressively more negative hypotheses that are drafted in to explain them away (this can also be put in terms of “circular inference,” see Jardri and Deneve, 2013).

In Type 2 trauma, what we have is a more general type of learning, over an extended period of time. The result of this is an impression, built up from statistical regularities in past experience, that the world is not generally a kind or safe place. What this does is lead to more general biasing toward threatening hypotheses, rather than the reliving of a very specific, rich, and multi-level hypothesis (as is the case with Type 1 trauma).

This means that events are given more negative and threatening interpretations than they warrant, which in turn leads to high levels of negative affect, of anxiety and hypervigilance. This in turn would lead to a vicious cycle since the propensity toward negative affect would bias perceptual inference in favor of negative hypotheses in the same way as the feedback loop mentioned above. A positive or neutral hypothesis explains only exteroceptive signals, but is actually at odds with interoceptive signals (with negative hedonic valence), namely, your negative emotional state. Your nervous system effectively “reasons”: “Why would I be afraid if this situation was innocuous?” A negative hypothesis on the other hand explains both exteroceptive and interoceptive signals. You instead get: “I’m afraid, therefore there must be something to be afraid *of*.” This goes some way toward explaining the hypervigilance and startle response that we see in individuals who have undergone Type 2 trauma.

Of course, the distinction between Type 1 and Type 2 trauma won’t always be clear-cut, and many people will present with symptoms of both. All that you would need for this symptomatic presentation is for a single event of cataclysmic significance to make one multi-level hypothesis have aberrantly high prior probability (Type 1 flashbacks) and for either that same event, or (more likely) an extended period of threat in addition to that one event, to lead to a general skewing of priors toward negative hypotheses, namely, to the learnt view that the world is not a safe place.

### PTSD vs. Trauma-Based Psychosis

It is well established that there is a strong statistical relationship between trauma and psychosis (see Morrison et al., 2003). How does psychosis differ from PTSD, and where do we draw the line between the two? The line may not be clear-cut in terms of the extent to which some cases of PTSD have elements of psychosis, but the concepts “PTSD” and “psychosis” are very different. Psychosis, in its purest sense, means a disconnection from reality. To the extent that one can be more or less disconnected from reality, psychosis comes in degrees; people can be more or less psychotic (in other words, psychosis can vary from mild to severe). On the other hand, someone can experience PTSD flashbacks, but recognize them as such, dismiss them as not telling them anything about the world (and rather as revealing something about their own mind, rather than the world) and hence will not be disconnected from reality. They will not, as a result, take themselves to inhabit a world that is different from the actual world. We take it that many cases of PTSD are non-psychotic in this sense. In psychosis, however, the person takes herself to be living in a world that is different from the actual world: she has an inaccurate world-view, is disconnected from reality. Now, of course, someone could, due to their past (i.e., involving trauma) and symptomatic presentation, have a diagnosis of PTSD, but also present with psychosis, namely, with some degree of disconnection from reality. There are two questions to address here.

(i) What does this amount to in predictive processing terms? And how does the difference between non-psychotic PTSD and post-traumatic psychosis play out?

(ii) What does the PPF tell us about how these two different cognitive states might arise?

The key to answering (i) lies in the hierarchical arrangement of hypotheses. “Lower” in the hierarchy, the hypotheses correspond to percepts. Thus, an inaccurate hypothesis at this level corresponds to hallucination or illusion. At higher “levels” what we are talking about are beliefs, more abstract reflections of the subject’s world-view. Hypotheses at the lower level don’t need to entail that hypotheses higher up fall into place: they can be, and often are, compartmentalized. The clearest example of this is in cases of optical illusions. You can measure the lines in the Muller-Lyer illusion to prove to yourself that they are the same length, but your visual system will still “believe” that they are different lengths. Here your high-level hypothesis (i.e., your belief) is that the lines are the same length, even though your visual experience, determined by a lower-level hypothesis, is telling you the contrary. To take an equivalent but more ecologically valid example, somebody can hear a voice, but dismiss that as merely the product of their nervous system (Nayani and David, 1996). Alternatively, they could update their world-view, and take there, for example, to be an autonomous agent who is communicating with them. The former case is not (contrary to what some diagnostic manuals might tell you) psychotic, whereas the latter is. Aberrant sensory experiences need not lead to full-blown psychosis. Furthermore, the intensity of the aberrant experiences needn’t correlate with the extent to which the experiences are taken seriously. This can be clearly seen in Charles Bonnet syndrome, where subjects have intense visual hallucinations, which they always recognize as “just in the head” and not real (Berrios and Brook, 1982).

Another factor is to do with the content of the hypotheses selected, which constitute these experiences, and the extent to which they can be connected to the past by the person having the experience. In Type 1 trauma the hypothesis (and hence the experience) is of a specific event from the subject’s past, and so there is less temptation to think that the flashback is part of the current world. In Type 2 trauma, the hypotheses that correspond to the symptomatic experiences are less specific and hence less obviously associated with the past, although the person may still be aware of the association, and hence also able to reject them as something that’s not happening in the here-and-now. In psychosis, the hypotheses tend not to be highly specific, tend not to be connectable to a distinct past event, and they tend to be about current experience. This capacity to appreciate whether the experience is about the past or the present, and how the specificity of the hypotheses in question is a factor in this, may go some way toward explaining why some people experience psychosis after trauma (Brewin, 2015). It also helps to explain why the line between PTSD and psychosis is blurred (Powers et al., 2016), and more likely to be blurred specifically between Type 2 rather than Type 1 trauma and psychosis.

In answer to (ii), we need to give an account of what is likely to lead to aberrant hypotheses higher up the hierarchy, rather than the sort of compartmentalized hypotheses we see in, for example, non-psychotic PTSD. This requires us to reflect on the kinds of trauma and, in particular, the way in which trauma will have different impacts at different stages of the life-course. More pervasive aberrations are more likely to be caused at times when basic priors are being laid down, namely, early in life when general and fundamental learning takes place. These basic priors may not determine specific factual beliefs, but rather *styles* of thinking; general statistical appreciation of what is a plausible inference to make. Trauma early in life, and especially at the hands of a primary care-giver, is likely to yield a basic lack of trust in the world (Herman, 1997). This should not be thought of as the acquisition of a *belief* about the world. Rather it should be seen as a background condition for the way in which beliefs will be formed^7^. To put it in the terminology of the PPF, it is about *priors* rather than specific hypotheses. Thus, aberrant sensations are more likely, due to this skewing of priors, to lead to aberrant beliefs: the low-level hypotheses, more likely to yield aberrant hypotheses at higher levels in the hierarchy.

## Predictive Processing and Existing Theories

It is important to clarify the scope of our suggestion. We do not view the PPF as a competitor to existing theories of PTSD. We see it as compatible with, and at times as a useful reconceptualization of, these theories. Although there are a multitude of such theories, the following three are arguably the most influential. We present them first, and then recast them in terms of the PPF in a way that we think proves to be illuminating.

### Conditioning Theories

Some of the earliest theories of PTSD built on theories of conditioned response. According to these theories, the initial traumatic event is the unconditioned stimulus, and the powerful reaction of distress to that event is the unconditioned response. This reaction leads to an “over-consolidation” of traumatic memories, and reminders become conditioned stimuli, with powerful and irrational fear responses being the conditioned response. Sometimes the reactions are so powerful that, beyond a straightforward fear response, there are also flashbacks.

The notion of conditioning falls very naturally out of the PPF [see Powers et al. (2017) for a study involving conditioning-induced hallucinations]. The PPF states that the nervous system uses its hierarchical Bayesian machinery to “plug into” the statistical structure of the world: and conditioning is just a form of statistical associative learning. But something doesn’t have to be presented lots of times in order to be given a high prior probability, since not all stimuli have the same importance, or the same valence. Something of extremely high importance, and high valence, is likely to both generate and be strongly associated with a strong experience of negative arousal (fear).

In short, conditioning theories are very much in keeping with the PPF. It is natural to view the PPF as simply providing the mechanism that underpins such conditioning. It is worth mentioning that these theories seem to be most applicable to Type 1 trauma.

### Emotional Processing Theories

Emotional processing theories (like the one proposed by Foa and Kozak, 1986) argue that “complex fear structures” are stored in memory and produce cognitive, behavioral, and physiological reactions when “activated.” Although these structures are important in generating adaptive reactions to danger they can become maladaptive in certain contexts. What happens in PTSD is that a benign stimulus becomes associated with danger and activates these complex fear structures. This can be thought of as a fleshed-out version of the conditioning account. Furthermore, it should be noted that, unlike the straightforward conditioning theory, emotional processing theories have more flexibility in being applicable to both Type 1 and Type 2 trauma.

This account is itself further elaborated when viewed from within the PPF. According to the PPF, whereas perceptual experience corresponds to the hypothesis about what is going on that best explains exteroceptive signals, emotional experience corresponds to the hypothesis about what is going on that best explains interoceptive signals (Seth, 2013). In actual fact, perceptual and emotional experience cannot be pulled apart: perception is shot through with affect, and our experiences (hypotheses) integrate a wealth of information both exteroceptive and interoceptive. But our nervous systems can learn, due to extreme circumstances, to over-react interoceptively to relatively benign stimuli. This will activate complex hypotheses about what is going on that aren’t simply fear, but will include perceptual and cognitive elements too.

### Cognitive Theories

Finally, there are cognitive theories like that of Ehlers and Clark (2000), which casts PTSD in terms of beliefs or appraisals. In short, PTSD sufferers develop excessively negative appraisals about both the external world and themselves: they view the world as a dangerous place, and they view themselves as incapable. Memory recall and fear responses become biased by these negative appraisals, and it is these appraisals that need to be targeted by talking therapies.

Again, such an account fits nicely within the PPF. The negative appraisals about the world and oneself may, however, take on a different structure within the PPF. A negative appraisal of the world could be seen, since it lacks the specificity of any given hypothesis, as the acquisition of priors in favor of negative hypotheses more generally. Negative self-appraisal, on the other hand, is to do with one’s self-conception. Within the PPF this would be viewed as the generative model for oneself, that remains largely stable over a long period of time, and which one carries with one and brings to bear upon every encounter. Viewing oneself as useless and incapable will bias our engagement with the world into fulfilling that view. Things that aren’t really challenging will be seen as such, things that aren’t really threatening will be seen as such, and so on. Of course, this has all sorts of emotional upshots that feed into and bias the lower-level machinery. This kind of holism is precisely the sort of thing that cognitive theories would embrace, and is especially useful for thinking about Type 2 trauma.

## Conclusion and Clinical Directions

We would expect Type 1 and Type 2 trauma to be responsive to different treatments. We also get a sense of what any such treatments would need to achieve if our account is accurate. In the Type 1 case, we need to lower the prior probability of the “trauma hypothesis.” This would be achieved, for example, through gradual exposure to the maladaptive triggers. In effect this would result in the subject re-learning the statistical relationship (a rather lack thereof) between the traumatic hypothesis and the triggers. In the Type 2 case, the nervous system’s faith in the world in general needs to be restored. Pathways to achieving this are perhaps less obvious. It would likely be a slower, more complex process. This is born out by the generally worse prognoses of Type 2 trauma, and the kinds of therapies that are effective (e.g., exposure therapy is highly effective in Type 1 cases, and much less effective in Type 2 cases). Another tactic that might be beneficial would be to target the interoceptive states that fuel the circular inferences we mentioned above. Thus, perhaps a treatment of underlying emotional states, such as anxiety, might be effective. These treatments could be pharmacological or talking therapy-based^8^. They may involve the construction, both in actual fact, and psychologically, of a “safe space.”

Since both exposure and the creation of a safe space involve the nervous system generating hypotheses, the richer such exposure or safe space is, the more effective the treatment will be. This suggests that immersive techniques such as Virtual Reality (VR) will be more effective than narrative or even recollective and imaginative exercises. These have been used effectively both for exposure therapy in the contexts of trauma (Botella et al., 2015) and in the creation of safe spaces in paranoia (Freeman, 2008). Although highly effective in the case of exposure, it remains to be conclusively established whether it is more effective than more traditional forms of exposure.

How would the PPF suggest we treat dissociation? It is not clear that peritraumatic dissociation is something that we can, or indeed need to, treat (since it is by definition short-lived). Post-traumatic dissociation, however, requires the hierarchy to be reunified. This would require some sort of attentional process (where, as we mentioned, this involves turning up the precision on prediction error), where the precision in the lower/mid-level of the hierarchy (wherever the “break” is) is restored. The performing of a basic, safe, and affectively neutral task may be helpful in this regard. EMDR is used in a wide variety of clinical contexts, and it might be effective here (some recommend it for dissociative disorders, see Knipe, 2008), as might something more mundane, like playing Tetris [which Iyadurai et al. (2017) have found reduces intrusive memories in trauma]. Indeed more generally future research within the PPF might, in unearthing the true mechanisms of therapeutic efficacy, demonstrate that some of the best treatments are simple, cheap, and require little training on the part of the therapist, and can therefore be widely and efficiently administered.

## Author Contributions

All authors listed have made a substantial, direct and intellectual contribution to the work, and approved it for publication.

## Conflict of Interest Statement

The authors declare that the research was conducted in the absence of any commercial or financial relationships that could be construed as a potential conflict of interest.

## Footnotes

1 These symptoms are often divided into three “symptom clusters,” namely, (i) intrusive phenomena, (ii) arousal symptoms, and (iii) avoidance symptoms.

2 Some theorists use the term “complex trauma” or “complex PTSD” in a way that is synonymous with “Type 2 trauma.” At other times it seems to refer to a combination of Type 1 and Type 2 symptoms.

3 We say that this is “schematically put” because each of the “levels” in the hierarchy represented in the diagram here are actually made up of countless “sub-levels,” which cannot be easily represented in words or diagrams.

4 In a related manner, this suggests that there is no clean distinction between traumatic and non-traumatic events: they are on a spectrum.

5 Nowhere is this more starkly illustrated than in the context of torture (Nickerson et al., 2016).

6 From a different perspective, this can be understood in terms of error-management theory (EMT) [see Dodgson and Gordon (2009) for an application of EMT to auditory hallucinations]. EMT effectively states that it is adaptive for an organism’s nervous system to err on the side of caution by biasing its reasoning in favor of false positives in matters pertaining to fitness (i.e., survival or reproduction).

7 In phenomenological term, this is akin to what Ratcliffe (2008) calls an “existential feeling.”

8 Indeed, there is some evidence that beta-blockers reduce the frequency and severity of flashbacks (van Stegeren, 2005).
